# Deficiency of Biogenic Amines Modulates the Activity of Hypoglossal Nerve in the Reserpine Model of Parkinson’s Disease

**DOI:** 10.3390/cells10030531

**Published:** 2021-03-02

**Authors:** Monika Jampolska, Kryspin Andrzejewski, Małgorzata Zaremba, Ilona Joniec-Maciejak, Katarzyna Kaczyńska

**Affiliations:** 1 Department of Respiration Physiology, Mossakowski Medical Research Institute, Polish Academy of Sciences, 02-106 Warsaw, Poland; mjampolska@imdik.pan.pl (M.J.); kandrzejewski@imdik.pan.pl (K.A.); 2 Department of Experimental and Clinical Pharmacology, Centre for Preclinical Research (CePT), Medical University of Warsaw, 02-091 Warsaw, Poland; mzaremba@wum.edu.pl (M.Z.); ijoniec@wum.edu.pl (I.J.-M.); 3 Laboratory of Experimental Therapies, Military Institute of Hygiene and Epidemiology, Kozielska 4, 01-163 Warsaw, Poland

**Keywords:** Parkinson’s diseases, biogenic amines, reserpine model, hypoxia, hypoglossal nerve, phrenic nerve

## Abstract

The underlying cause of respiratory impairments appearing in Parkinson’s disease (PD) is still far from being elucidated. To better understand the pathogenesis of respiratory disorders appearing in PD, we studied hypoglossal (HG) and phrenic (PHR) motoneuron dysfunction in a rat model evoked with reserpine administration. After reserpine, a decrease in the baseline amplitude and minute HG activity was noted, and no depressive phase of the hypoxic ventilatory response was observed. The pre-inspiratory time of HG activity along with the ratio of pre-inspiratory time to total respiratory cycle time and the ratio of pre-inspiratory to inspiratory amplitude were significantly reduced during normoxia, hypoxia, and recovery compared to sham rats. We suggest that the massive depletion of not only dopamine, but above all noradrenaline and serotonin in the brainstem observed in our study, has an impact on the pre-inspiratory activity of the HG. The shortening of the pre-inspiratory activity of the HG in the reserpine model may indicate a serious problem with maintaining the correct diameter of the upper airways in the preparation phase for inspiratory effort and explain the development of obstructive sleep apnea in some PD patients. Therapies involving the supplementation of amine depletion other than dopamine should be considered.

## 1. Introduction

Parkinson’s disease (PD) is the second most common neurodegenerative disorder, after Alzheimer’s disease, associated with substantial damage of dopaminergic neurons in the nigrostriatal pathway that is responsible for the characteristic motor symptoms of the disease [1,2,3]. Other underestimated PD-related symptoms are respiratory impairments such as dysrhythmic breathing pattern, dyspnea, decreased respiratory pressure, and sleep-disordered breathing [4,5,6]. The underlying cause is still far from being elucidated. It is suspected that respiratory disturbances may be associated with deficits in two other monoamine systems, such as noradrenergic and serotoninergic systems, that apart from dopaminergic, are depleted in PD brains [7,8,9,10,11]. All these monoamines are transmitters of neurons present in structures important for the generation and modulation of the respiratory rhythm or neurons localized in their immediate vicinity [10,12,13,14,15,16,17,18].

It is generally acknowledged that the reduced release of noradrenaline (NA) and serotonin (5-HT) during sleep leads to reduced excitability of hypoglossal (HG) motoneurons [19,20,21,22]. All the extrinsic and intrinsic muscles of the tongue, except for the palatoglossus, are innervated by the hypoglossal nerve that is involved in controlling tongue movements required for speech as well as swallowing, and maintaining patency of the upper respiratory tract [22]. Proper contraction of the upper airway muscles and tongue is needed to protect the airway from collapse during sleep. Dysfunction in the HG nerve control of genioglossus muscle tension may result in a blockage of the respiratory tract at the throat level contributing to the patomechanism of obstructive sleep apnea (OSA) [23]. Interestingly, OSA prevalence in PD subjects has been demonstrated in several studies [24,25,26].

A rat reserpine model of PD is well-known to produce a wide range of motor impairments (akinesia, hypokinesia, limb rigidity, oral tremor) as well as affective disorders (memory deficits, depressive, anxiety, and anhedonic-like behaviors) that resemble PD [27,28,29,30]. Reserpine is a specific inhibitor of the vesicular monoamine transporter (VMAT2) that induces a loss of storage capacity and extensive depletion of brain DA, 5-HT, and NA [31,32]. A substantial shortage of all these monoamines in the brainstem structures that are involved in the regulation of breathing may have an impact on respiratory impairments reported in PD, including OSA. An investigation of how the substantial depletion of DA, 5-HT, and NA affects HG nerve activity in the latter model may be relevant for increasing knowledge of the pathophysiology of OSA in PD.

Therefore, the goal of the present study was to examine whether and how a substantial depletion of all three monoamines, DA, 5-HT, as well as NA, in the brainstem, impact the neural respiratory activity to the upper respiratory muscles and the diaphragm, the main respiratory muscle. To study the activity of the hypoglossal nerve and phrenic nerve (PHR), providing the only motor supply to the diaphragm, we used the reserpine model of parkinsonism in which we confirmed the extensive depletion of all test monoamines in both striatum and brainstem. Nerve activities were investigated during normoxia and following a well-known stressor, acute hypoxia.

## 2. Materials and Methods

### 2.1. Animals and Compounds Used to Induce Parkinsonism

This study was conducted according to the guidelines of the Declaration of Helsinki and approval by the Local Warsaw Ethics Committee (WAW2/134/2018 approved on 21 September 2018).

The study was conducted on 16 male adult Wistar rats, weighing 270–300 g (8–10 weeks old). The rats were divided into 2 groups:

1. In the first group (*n* = 7), the model of PD was induced using reserpine (Sigma Aldrich, Poznań, Poland) and α-methyl-p-tyrosine (AMPT) (Sigma Aldrich, Poland).

2. The second group of rats created sham animals that received vehicle injections (*n* = 9).

Reserpine, dissolved in a mixture solution consisting of 0.25% citric acid, 2% benzyl alcohol, and 10% Tween-80, was administered intraperitoneally (i.p.) at a dose of 2.5 mg/kg. Seventeen hours after the injection of reserpine, AMPT dissolved in saline was administered intraperitoneally in a dose of 250 mg/kg. AMPT is a tyrosine hydroxylase enzyme inhibitor that inhibits the synthesis of DA and NA and prolongs the neurochemical deficits.

### 2.2. Electrophysiological Experiments

**Figure 1:**
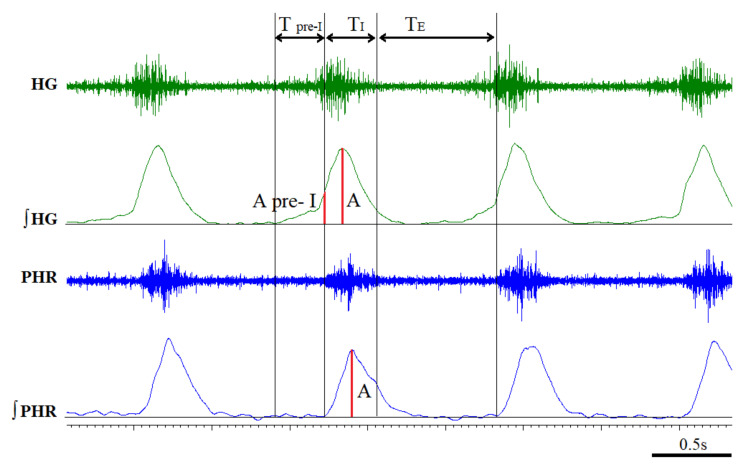
Sample record of neurograms of the hypoglossal nerve (HG, XII nerve) and the phrenic nerve (PHR) showing the calculation method of the following parameters: inspiratory time (T_I_), expiratory time (T_E_), pre-inspiratory time of HG nerve activity (T pre-I), the amplitude (A) of HG and PHR, amplitude of HG pre-inspiratory activity (A pre-I HG).

Nineteen hours after an i.p. injection of reserpine (Sigma Aldrich, Poland) or vehicle, and two hours after an AMPT (Sigma Aldrich, Poland) i.p. injection, the animals were anesthetized intraperitoneally with 750 mg/kg of urethane (Sigma Aldrich, Poland) and 150 mg/kg of α-chloralose (Fluka, Munich, Germany). The animal’s femoral artery, to monitor blood pressure, and the femoral vein, to administer supplemental anesthesia and fluids, were cannulated. Rectal temperature was monitored and maintained throughout the experiment at 37–38 °C via an external heating pad. Arterial blood pressure was measured with a BP-2 Columbus Instruments (Columbus, OH, USA). After a tracheostomy performed to allow artificial ventilation, the rats were treated with muscle relaxant; pipecuronium bromide (Arduan, Gedeon-Richter, Budapest, Hungary) given at a dose of 0.08 mg/kg. Afterwards, the animals were artificially ventilated with a rodent ventilator (7025 Ugo Basile, Gemonio VA, Italy) attached to the tracheostomy tube. End-tidal CO_2_ was measured (Capstar-100, CWE Inc., Ardmore, PA, USA) and kept between 4.5 and 5.0%. Both cervical vagi were transected at the mid-cervical level and cut to abolish the regulation of the respiratory activity along with the inflation of the lungs caused by the respiratory pump. The central ends of the whole phrenic nerve and the main hypoglossal trunk, after transection in the neck, were arranged on bipolar silver electrodes for nerve activity recording. The recorded nerve activity was amplified, filtered (5–2500 Hz), and rectified with a NeuroLog system (Digitimer Ltd., Wewelyn, UK). Data acquisition interface (CED Power 1401) was used to digitize raw and integrated nerve activities, recorded and analyzed with Spike 2 software (Cambridge Electronic Design, Cambridge, UK). The acute hypoxia experimental protocol was ventilation with 8% oxygen in nitrogen. Each exposure to hypoxia lasted 1.5 min or was interrupted when an apnea episode occurred. All analyzed data were calculated based on integrated phrenic and hypoglossal neurograms, as previously described [33,34]. Ventilatory parameters like amplitude and frequency were calculated for each nerve as shown in Figure 1.

Frequency (f), inspiratory time (T_I_), the expiratory time (T_E_), and the respiratory cycle time (T_C_) were calculated from the phrenic nerve activity. The beginning of T_I_ was set as the start of the increase of integrated activity of the phrenic nerve. The end of T_I_ was determined at the point of a 50% decrease of the maximal amplitude of the phrenic nerve. T_E_ was defined as a time between the end and start of T_I_. T_C_ consisted of T_I_ and T_E_. The time point adequate to the onset of phrenic nerve activity appointed the amplitude of the pre-inspiratory hypoglossal activity (A pre-I HG). The length of the pre-inspiratory hypoglossal activity (T pre-I HG) was computed as the time period between the start of the integrated hypoglossal activity and the onset of phrenic nerve activity. All parameters were analyzed at the baseline, in maximum nerve activity (maximum amplitude during the hypoxic episode), and 30 s after apnea of each hypoxic episode (recovery). Changes in amplitude, frequency, and minute activity were expressed in absolute values nerve activity and reported as the means ± SEM. The duration of the T pre-I HG was calculated relative to the duration of the total time of the respiratory cycle (T pre-I/Tc), while the amplitude of the pre-I HG was presented as a fraction of the peak inspiratory hypoglossal amplitude (A pre-I HG/A HG).

### 2.3. High-Performance Liquid Chromatography (HPLC) Analysis. Assay of Dopamine, Serotonin, Noradrenaline, and Its Metabolites

Following electrophysiological experiments, the animals were euthanized with an overdose of pentobarbital sodium and their brains were immediately removed. Both striatum and brainstem were dissected from the left and right sides. Each tissue sample was weighed and frozen (−80 °C) until further biochemical analysis.

Brain tissue was sonicated in ice-cold 0.1 M HClO4 solution with 0.05 mM ascorbic acid and centrifuged (13,000 rpm, 15 min at 4 °C) to precipitate proteins. The supernatant was filtered (0.2 μm pore size filter; Whatman, Chicago, IL, USA). The total tissue content of DA, 5-HT, NA and their analogous metabolites: 3, 4-dihydroxyphenylacetic acid (DOPAC), homovanillic acid (HVA), 5-hydroxyindolacetic acid (5-HIAA), and 3-methoxy-4-hydroxyphenylglycol acid (MHPG) were measured by HPLC-ED assay with L-3500 electrochemical detector (Merck, Darmstadt, Germany) containing a glassy carbon electrode. A voltage was set at 0.8 V and an Ag/Ag Cl reference electrode. Samples of 20 µL were added into a C-18 reverse phase column (250 × 4.6 mm Macherey-Nagel, Duren, Germany) with Nucleosil 5 μm particle size. The mobile phase was dissolved in 18.3 mΩ purified water with 12% CH3OH (Merck, Germany), and the composition of the mobile phase was as follows: 32 mM NaH2PO4 (Sigma-Aldrich, St. Louis, MO, USA), 34 nM C6H8O7 (Sigma-Aldrich, USA), 1 mM C8H17NaO3S (Sigma-Aldrich, USA), and 54 μM EDTA (Sigma-Aldrich, USA). The infused flow rate was set at 0.8 mL min^−1^.

Samples were quantified by comparing them with the standards (Sigma-Aldrich, USA) using external standard calibration ClarityChrom software (Knauer, Berlin, Germany). The contents of monoamines and parallel metabolites were expressed as pg mg^−1^ of fresh tissue.

### 2.4. Statistics

A Shapiro-Wilk test was performed to determine if the values tested were within the normal distribution. One-way ANOVA was used for comparison within the group between baseline and defined time points during and after hypoxia. Differences between individual time points and experimental conditions (sham vs. reserpine) were evaluated by Student’s *t*-test for independent samples. The data were analyzed with STATISTICA 12 (StatSoft Polska, Kraków, Poland) and presented as means ± SEM. In all cases, *P* ≤ 0.05 was considered statistically significant.

## 3. Results

### 3.1. Changes in the Normoxic and Hypoxic Activity of the Hypoglossal (HG) and Pphrenic (PHR) Nerves in Reserpine and Sham Rats

**Figure 2:**
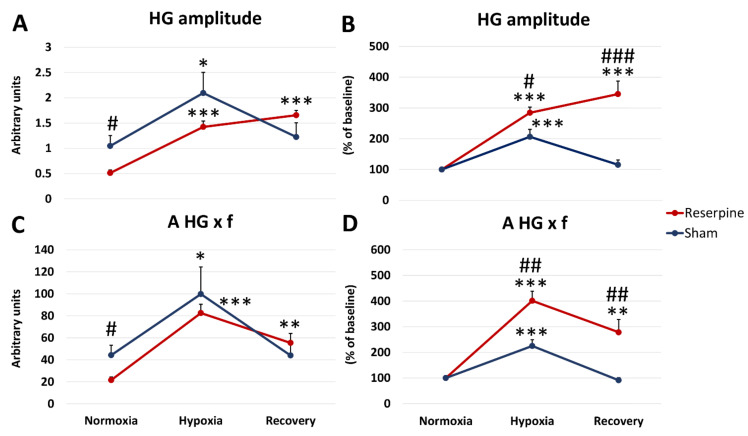
The average amplitude (**A**,**B**) and minute activity (A × f) of the hypoglossal nerve (HG) (**C**,**D**) during normoxia, hypoxic ventilatory response, and recovery in animals subjected to a vehicle (sham) or reserpine injection. Results are expressed as absolute values (**A**,**C**) and a percentage of the baseline nerve activity (expressed as 100%) (**B**,**D**) before hypoxia. All values are means ± SEM. *# *P* < 0.05, **## *P* < 0.01, ***### *P* < 0.001,*—statistical significance in comparison to normoxic value, #—statistical significance between corresponding values in reserpine and sham groups (*n* = 7–9 per group).

In reserpine rats during air-breathing, the amplitude of the hypoglossal nerve was significantly decreased compared to the sham group (Figure 2A).

During a ventilatory response to acute 8% (O_2_ in N_2_) hypoxia (HVR), the HG nerve amplitude in the reserpine group increased three times compared to its baseline value, while in sham rats only twice (Figure 2B). Despite this, it did not reach the level of magnitude present in the sham group with absolute values (Figure 2A). During recovery breathing after apnea, the amplitude of HG in the reserpine rats increased further compared to the hypoxic response, while sham rats showed almost return to the baseline pre-hypoxic value (Figure 2A,B). This time absolute values of amplitude were higher in the reserpine group, although insignificant (Figure 2A).

During normoxia, the magnitude of minute activity of HG (A HG × f), was twice lower in the reserpine-treated rats. The parameter increased in a significant manner during hypoxia in both groups; during recovery it returned to baseline in the sham group, while in the reserpine rats it was twice significantly increased in comparison to its baseline (Figure 2C). When expressed as a percentage of change, A HG × f in the reserpine rats was augmented four times during hypoxia, while in the sham group twice only (Figure 2D).

**Figure 3:**
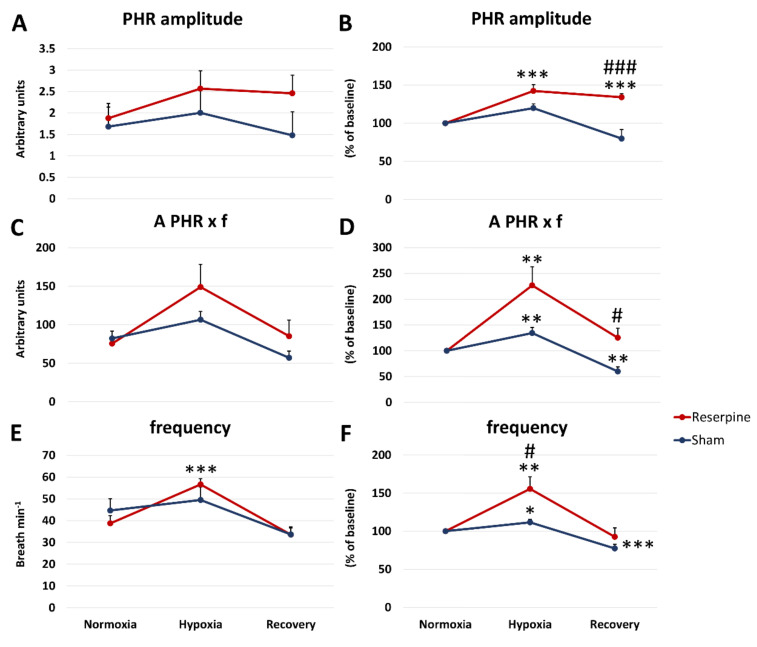
The changes in amplitude (**A**,**B**), minute activity (A × f) (**C**,**D**), and frequency of discharge (f) (**E**,**F**) of the phrenic nerve (PHR) during normoxia, hypoxic ventilatory response, and recovery in animals subjected to a vehicle (sham) or reserpine injection. Results are expressed as absolute values (**A**,**C**,**E**) and percentage of the baseline nerve activity (considered 100%) (**B**,**D**,**F**) before hypoxia. All values are means ± SEM. *# *P* < 0.05; **## *P* < 0.01, ***### *P* < 0.001, *—statistical significance in comparison to normoxic value, #—statistical significance between corresponding values in reserpine and sham groups (*n* = 7–9 per group).

The amplitude and minute activity of the phrenic nerve were not significantly different between the two examined groups during normoxic breathing (Figure 3A,C).

During the ventilatory response to hypoxia, both parameters of the PHR nerve were higher in the reserpine state, although not significantly in comparison to the sham group when expressed as a percentage of its baseline values (Figure 3B). The values observed during the recovery time after apnea and hypoxia were also higher and reached statistical significance in the reserpine rats (Figure 3B,D).

### 3.2. Time Components of HG and PHR Respiratory Activity during Normoxia and Hypoxia Response in Reserpine and Sham Rats

There were no significant differences between compared groups of rats in the alteration of the frequency of nerve discharges (f), which was the same for both examined nerves during normoxic breathing and the ventilatory response to hypoxia (Figure 3E). However, compared to the control group, reserpine rats showed a significantly magnified increase in f during HVR expressed as a percentage of change from the baseline (Figure 3F).

**Figure 4:**
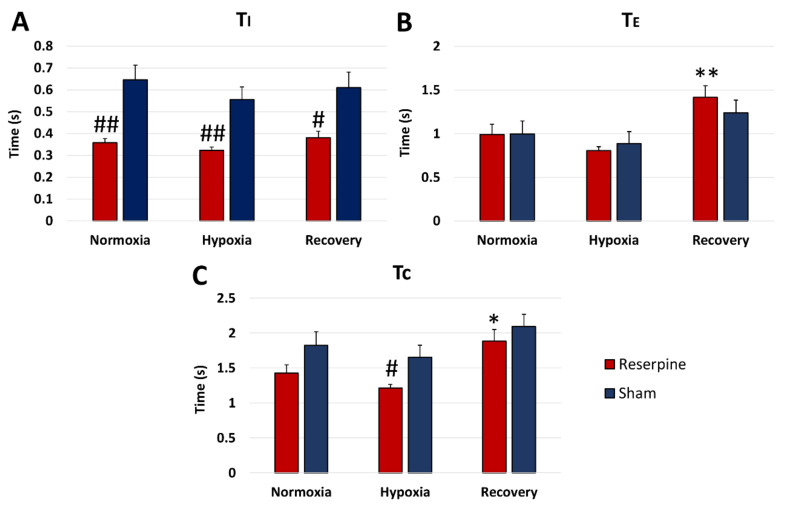
The changes in time of inspiration (T_I_) (**A**), time of expiration (T_E_) (**B**), and the total time of respiratory cycle (T_C_) (**C**) in the respiratory response to hypoxia in rats injected i.p. with vehicle (sham) or reserpine. All values are given as mean ± SEM. *# *P* < 0.05; **## *P* < 0.01, *—statistical significance in comparison to normoxic value, #—statistical significance between corresponding values in reserpine and sham groups (*n* = 7–9 per group).

Unlike the sham group, the reserpine rats showed significantly shortened T_I_ during normoxia, hypoxia, and recovery (Figure 4A). This translated into a statistically significant diminished total time of respiratory cycle (T_C_) of the reserpine rats during HVR only (Figure 4C). We did not find the difference between groups in the time of expiration (T_E_) parameter. It was only significantly extended during recovery time, together with Tc in the reserpine state compared to the control values (Figure 4B).

An analysis of the duration of hypoxia to apnea revealed that the sham rats achieved apnea after 55 ± 7 s, which lasted about 56 ± 9 s. The reserpine rats achieved apnea after an average 60 ± 7 s, and the arrest of breathing lasted 42 ± 5 s. Comparing any of these parameters showed no statistical significance when making comparisons between the groups (*P* = 0.6 for a time of apnea appearance and *P* = 0.17 for a time of apnea).

### 3.3. The Pre-Inspiratory Activity of the Hypoglossal Nerve in Reserpine and Sham Rats

**Figure 5:**
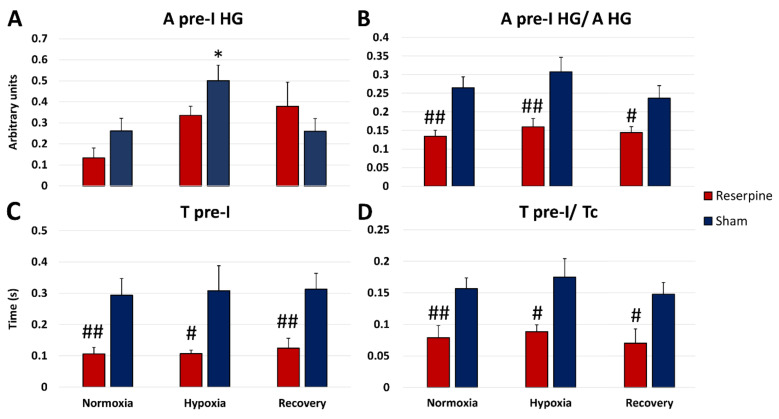
The average pre-inspiratory hypoglossal nerve amplitude (A pre-I HG) (**A**), a ratio of the pre-inspiratory hypoglossal nerve amplitude to the inspiratory HG peak amplitude (A pre-I HG/A HG) (**B**), pre-inspiratory time of HG (T pre-I) (**C**) and the ratio of pre-inspiratory time of HG to a total length of the respiratory cycle (T pre-I/Tc) (**D**) in respiratory response to hypoxia in rats injected i.p. with vehicle (sham) or reserpine. All values are given as mean ± SEM. All values are given as mean ± SEM. *# *P* < 0.05; ## *P* < 0.01, *—statistical significance in comparison to normoxic value, #—statistical significance between corresponding values in reserpine and sham groups (*n* = 7–9 per group).

In both groups, the pre-inspiratory amplitude of HG (A pre-I HG) was raised at the hypoxic peak in comparison to the baseline values; however, only the sham rats reached the significant value (Figure 5A). In reserpine rats, during recovery, this was insignificantly increased, while sham returned to a normoxic level. There was no significant difference in A pre-I HG value in normoxia, hypoxia, and recovery between both examined groups (Figure 5A).

**Figure 6:**
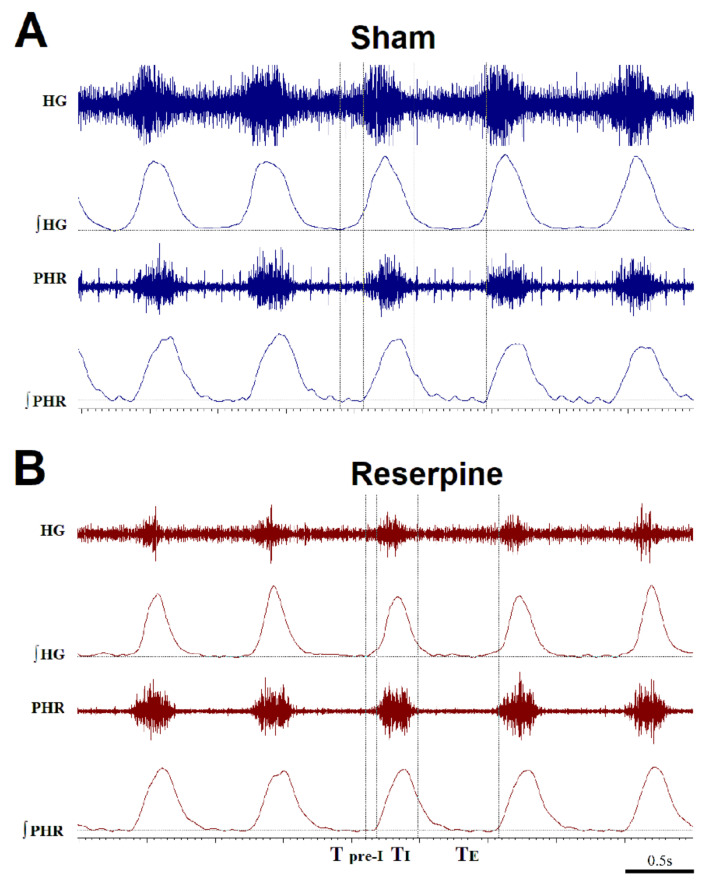
Typical neurogram records of phrenic (PHR) and hypoglossal (HG) nerve activity (raw and integrated signals—∫) in sham (**A**) and reserpine (**B**) rats during hypoxia. Note the apparently shortened time of inspiration (T_I_) and pre-inspiratory time of HG nerve activity (T pre-I) in the reserpine rats compared to the sham animals.

Comparing the reserpine and sham groups, the ratio of the pre-inspiratory amplitude to the amplitude of HG (A pre-I HG/A HG) in the parkinsonian group was significantly reduced during normoxia, HVR, and recovery (Figure 5B). A detailed analysis of the neural activity of the HG nerve showed that the T pre-I average value in reserpine rats was two and a half times less than in the sham rats during all examined conditions (Figure 5C and Figure 6). Likewise, the ratio of the time of pre-inspiratory activity to the total time of the respiratory cycle (T pre-I/Tc) was twice as low in the reserpine rats compared to the sham group, which was significant during normoxic, hypoxic, and recovery breathing (Figure 5D).

### 3.4. Content of Monoamines and Metabolites in the Striatum and Brainstem

The concentration of monoamines and their metabolites in both the striatum and brainstem of the sham, as well as the reserpine-treated rats, is presented in Table 1. In both investigated brain regions in the reserpine rats, we observed a substantial decrease in DA content; more than 90% in the striatum and 75% in the brainstem. The intermediate DOPAC and final HVA metabolites of DA were also significantly reduced; more than 90% in the striatum and 80% in the brainstem. The ratio of HVA/DA was significantly decreased only in the brainstem, which may prove that the compensation capacity of the DA system of this structure is smaller than in the striatum, and DA turnover shows a diverse regional pattern (Table 1). The striatal level of 5-HT decreased by 33%, while 5HIAA metabolite increased by 22%, which corresponds to twice the highest ratio of 5HIAA/5-HT in the reserpine than in the sham rats. In the brainstem of the reserpine rats, we noticed more intense changes in the serotonergic system than in the striatum; a 72% decrease in 5-HT and 28% increase in its metabolite 5HIAA level, and consequently a ratio of 5HIAA/5-HT that was about six times higher. Compared to the sham group, in the reserpine rats, the concentration of NA was decreased to a greater extent in the brainstem, by 92%, and to a lesser extent in the striatum, by 59%. The level of MHPG, NA metabolite was below the level of detection by our assays.

## 4. Discussion

We found that a substantial disruption of the bioavailability of three biogenic amines (DA, NA, and 5-HT) that modulate respiration significantly affects the activity of vital respiratory motoneurons in a non-invasive, repetitive model of PD, widely used as a predictive indicator of likely symptomatic efficacy of new agents [31].

The nerve whose activity changed more significantly was the hypoglossal nerve (HG, XII nerve) innervating the muscles of the upper airway, which are essential not only for swallowing and vocalization but also for breathing. The crucial role of the HG nerve is to prevent upper airway collapse by regulation of tongue position and stiffness, especially during sleep [22,35,36]. The disturbed activity of the hypoglossal motoneurons contributes to the development of obstructive sleep apnea syndrome (OSA) [21,37]. In support of this, numerous scientific reports showed that stimulation of the HG nerve in humans and animals has the benefit of a reduced number of pauses in breathing during sleep [38,39,40].

To better understand the pathogenesis of respiratory disorders appearing in PD and parkinsonism, we decided to study HG and PHR motoneuron dysfunction in an animal model evoked with reserpine administration.

The main finding was a significant reduction of the basal values of HG burst amplitude and HG minute activity (A HG × f). The latter was the effect of an amplitude decrease only since there was no difference in the frequency of nerve discharges between both states. Such meaningful changes in the normoxic activity of the HG nerve have not been observed in any of the previous studies in the unilateral [33,41] and bilateral [34] 6-OHDA models of Parkinson’s disease. This may be due to the significant differences in the brainstem loss of biogenic amines observed in individual models. Although 6-OHDA administration causes a significant decrease in levels of DA, 5-HT, and NA in the striatum, it has a limited effect on changes in the concentration of all amines in the brainstem [34,42,43]. For example unilateral medial forebrain bundle (MFB) injection caused a 20% decrease of 5-HT [43], while intracerebroventricular application evoked a 28% reduction of NA [34]. No changes in the DA concentration in the brainstem were observed in the aforementioned studies. On the other hand, in the present study reserpine produced a massive depletion of all amines in the brainstem-housing neuronal network that generate and control respiration.

The only study of respiration in a reserpine model to date has been conducted on conscious animals in a plethysmography chamber [44]. The animals presented diminished ventilation at rest and in response to hypoxia, which was restored after L-DOPA supplementation. It cannot be ruled out that the extreme stillness and muscle stiffness exhibited by the animals could be the cause of respiratory rate decline, in particular. To avoid the influence of behavior and dysfunction of the chest respiratory muscle pump on respiration, we used anesthetized and paralyzed animals (with the neuromuscular loop open) that were artificially ventilated.

During the hypoxic ventilatory response (HVR), the HG of reserpine rats, although taking off from a lower resting amplitude, was able to increase the value almost three-fold, while in sham rats only two-fold; nevertheless, the amplitude of the reserpine rats did not reach the absolute level of the sham group. The increased reactivity of the HG burst amplitude to hypoxia of the reserpine rats is in line with previous findings in 6-OHDA PD models [33,34]. A likely explanation for the similarity is the substantial depletion of striatal DA, a neurotransmitter that in normal conditions has been shown to inhibit the HVR when administered into the cerebral ventricle [45].

It appears that the loss of DA in the brainstem can affect the activity of the HG nerve as well. In the central nervous system, the presence of DA has been found in almost all regions of the brain respiratory neuron network [13,16,46], including structures sensitive to changes in the partial pressure of CO_2_ and pH [14], and the nucleus of the solitary tract (NTS) [47]. The application of DA agonists has been shown to inhibit spontaneously active neurons within the HG nerve nucleus, [48] and the D_2_ receptors, ubiquitous in the brain, have been located in the NTS and the motor nuclei of the medulla [49].

Interestingly, the increased release of DA from afferent chemoreceptive fibers in the NTS of a rabbit under the influence of a hypoxic stimulus was described by Goiny et al. [8] and attributed to DA contribution to the control of the depressive phase of the hypoxia response characterized by a pronounced phase of depression in phrenic nerve activity. The depressive phase of HVR is equivalent to our recovery time following hypoxia exposure and apnea presence. Therefore, the DA loss in the brainstem reported in our reserpine rats corresponds somewhat with an increased burst amplitude of HG and PHR during the recovery time and the absence of the depressive phase of the hypoxia.

One of the most affected parameters of the HG nerve in the reserpine model was its pre-inspiratory activity, which was significantly reduced in terms of duration (T pre-I, T pre-I/Tc) along with the inspiratory time (T_I_) under air-breathing, hypoxia, and post-hypoxic recovery. The pre-I HG nerve amplitude, although doubled during hypoxia, did not differ significantly between the groups. Nevertheless, the ratio of the pre-inspiratory amplitude to the inspiratory amplitude (A pre-I HG/A HG) in each hypoglossal outburst was significantly reduced compared to the sham group throughout the recording. As previously described [50], hypoxia augments the pre-inspiratory component of HG activity more than the inspiratory one, which means that the relative increase in amplitude is greater for pre-I. In our study this pattern was not maintained in the reserpine rats solely.

The period that occurs between the onset of hypoglossal and phrenic neural discharge constitutes the pre-inspiratory phase [51], responsible for the maintenance of the upper airway patency in preparation for the inspiration [50,52,53,54]. Consequently, the shortening of the pre-inspiratory activity of the HG nerve in the reserpine model indicates a serious problem with maintaining the proper diameter of the upper airways in the pre-inspiratory phase. This, in turn, may explain the reasons behind the development of OSA in some PD patients.

The problem of the increased frequency of episodes of apnea in Parkinson’s disease is still unclear. Scientific publications report similar [55,56] or increased risk [24,25] compared to the control population. The differences may be related to the stage of the disease and the individual pattern of pathological changes in the brain. The degeneration of the substantia nigra neurons and depletion of striatal DA are usually considered as the primary causes for PD [1,57]. Meanwhile, growing lines of evidence have shown that the noradrenergic neurons of the locus coeruleus (LC) also degenerate in the disease [11,57]. Moreover, the loss of NA neurons may be greater than that of DA neurons and even precede it [58,59]. Some studies indicate that the advancement of motor and non-motor symptoms may be correlated with the simultaneous loss of two monoamines: DA and NA [11,60].

NA is a well-known potent stimulus for breathing in normoxia, hypoxia, and hypercapnia [14,15,61,62]. As confirmation, awake rats with eliminated bulbospinal catecholaminergic C1 and A5 cells presented an impaired ventilatory response to hypoxia [63].

Pontomedullary sources of NA in the subcoeruleus, A5 and A7, noradrenergic regions send their projections to the hypoglossal motor nucleus [64]. What is more, pontomedullary premotor neurons with axonal projections to the HG nucleus are also targeted by NA [22]. Since NA induces an excitatory effect on HG motoneurons via α_1_-adrenergic receptors [65], its more than 90% reduction in the brainstem of reserpine rats may have contributed to a significant decline in the basal HG nerve amplitude. Unfortunately, the involvement of NA is more complex because, like 5-HT, such neurotransmitters mediate a wakefulness-related activation of HG motoneurons that is gradually withdrawn during sleep [22]. The study in anesthetized animals is a limitation of our research, which makes it more difficult to interpret the impact of NA and 5-HT brainstem deficiency on the activity of HG motoneurons. On the other hand, we wanted to avoid the influence of movement disorders on breathing; mainly the strong akinesia observed after reserpine in wakefulness. Therefore, a hypoxic stimulus that commonly activates NA, 5-HT, and DA neurons and stimulates respiration was applied.

The other nerve cells that degenerate or show dysfunction in Parkinson’s disease are 5-HT producing raphe nuclei neurons [66,67,68]. 5-HT has been described to mediate a key function in the body such as respiration [18]. In the dorsomedial medulla oblongata containing NTS and the nucleus of HG nerve, the hypoxic release of 5-HT stimulated airway dilation during hyperventilation and subsequent hypoxic ventilatory decline via 5-HT_2_ receptors [69]. 5-HT_2_ receptors play an extremely vital role in modulating the activity of the HG nerve by stimulating its motoneurons [70,71,72]. Thus, in the reserpine group, the significant loss of 5-HT in the brainstem, which could stimulate 5-HT_2_ receptors, corresponds to the lack of the depressive phase of the HVR, while the shortened values of the pre-inspiratory activity of the HG nerve may indicate a problem with upper airway patency.

## 5. Conclusions

To summarize, our study showed that the massive brainstem depletion of DA, NA, and 5-HT monoamines, which are of great importance in modulating the respiratory pattern during normoxia and hypoxia, has a strong influence on the pre-inspiratory activity of the hypoglossal nerve. The hypoglossal nerve dysfunction observed in the reserpine model of parkinsonism sheds new light on the cause of OSA reported in patients with neurodegenerative disease such as PD. Therapies based on the supplementation of amines depletion other than DA alone should be considered.

## Figures and Tables

**Table 1:** Comparison of the amount of biogenic amines in the striatum and brainstem between reserpine and sham rats. The concentration of dopamine (DA), noradrenaline (NA), serotonin (5-HT), 3,4-dihydroxyphenylacetic acid (DOPAC), homovanillic acid (HVA), and 5-hydroxyindolacetic acid (5-HIAA) was assessed by HPLC detection ex vivo and expressed as pg mg^−1^ of fresh tissue.

	DA	DOPAC	HVA	DOPAC/DA	HVA/DA	NA	5HT	5HIAA	5HIAA/5HT
*STRIATUM*									
Sham	11301	2791	1486	0.250	0.132	107.2	294.4	333.2	1.168
	± 678.3	± 206.5	± 119.7	± 0.019	± 0.008	± 4.172	± 28.19	± 38.70	± 0.140
Reserpine	764.24	201.61	85.24	0.372	0.214	44.01	197.3	407.6	2.309
	± 341.72	± 63.00	± 34.07	± 0.068	± 0.102	± 6.710	± 20.13	± 36.59	± 0.366
Student’s *t*-test	***	***	***			***	**		**
*BRAINSTEM*									
Sham	72.57	34.42	11.64	0.466	0.157	389.02	464.25	261.33	0.562
	± 3.289	± 4.708	± 1.751	± 0.050	± 0.019	± 16.03	± 13.18	± 15.530	± 0.028
Reserpine	17.64	6.015	1.294	0.318	0.040	30.30	127.43	334.22	3.775
	± 6.561	± 2.919	± 1.074	± 0.067	± 0.018	± 8.400	± 26.617	± 28.312	± 0.801
Student’s *t*-test	***	***	***		***	***	***	*	***

The data are expressed as mean ± SEM. * *P* < 0.05, ** *P* < 0.01, *** *P* < 0.001—significance between both groups (*n* = 7–9).

## Data Availability

The data used to support the findings of this study are available from the corresponding author upon request.
